# Leaf Functional Traits of *Caragana microphylla* Linked to Soil Microbial Communities Along a Restoration Chronosequence in Alpine Sandy Land

**DOI:** 10.3390/plants15193006

**Published:** 2026-09-30

**Authors:** Guilin Cao, Jinlu Guo, Wenjing Li, Yuan Wang, Qunce Chang, Lei Deng, Xuebin Zhao, Xiuben Yang, Zhe Chen, Shixiong Wang, Xiaogang Li, Wenying Wang, Jinhong Guan

**Affiliations:** 1 Key Laboratory of Surface Processes and Ecological Conservation of the Qinghai-Tibetan Plateau, College of Life Sciences, Qinghai Normal University, Xining 810008, China; gcaoguilin@163.com (G.C.); 18331825364@163.com (J.G.); wenjing010412@gmail.com (W.L.); 17782844473@163.com (Y.W.); quncec@163.com (Q.C.); chenzhe@qhnu.edu.cn (Z.C.); wangshix812@163.com (S.W.); wangwy0106@163.com (W.W.); 2 Key Laboratory of Biodiversity Formation Mechanism and Comprehensive Utilization of the Qinghai-Tibetan Plateau in Qinghai Province, Xining 810008, China; 3 Academy of Plateau Science and Sustainability, People’s Government of Qinghai Province and Beijing Normal University, Xining 810008, China; 4 College of Coastal Agricultural Sciences, Guangdong Ocean University, Zhanjiang 524088, China; pebbledeng@sina.com; 5 Qinghai Provincial Desertification Control Experimental Station, Gonghe 813000, China; 17716068272@163.com (X.Z.); 13897348807@163.com (X.Y.); 6 College of Ecology, Lanzhou University, Lanzhou 730000, China; lixiaogang@lzu.edu.cn

**Keywords:** restoration years, leaf functional traits, soil microbes, synergistic relationship, adaptive strategies

## Abstract

Vegetation restoration is accompanied by changes in plant functional traits, soil properties, and microbial communities, yet how these components covary along long-term restoration chronosequences remains poorly understood. We investigated *Caragana microphylla* plantations with 5, 10, 15, 23, and 36 restoration years in alpine sandy ecosystem to examine variations in leaf functional traits, soil properties, and soil microbial communities, as well as their associations during vegetation restoration. Leaf functional traits exhibited pronounced stage-dependent variation rather than simple linear patterns across the restoration chronosequence. During the early-to-middle restoration stages, *C. microphylla* exhibited relatively higher LTD and LDMC but lower SLA and LNC, with the most pronounced conservative trait combination observed at 15 Y of restoration. With later restoration stages, increased SLA and LNC at later stages suggested a tendency toward more acquisitive resource-use characteristics. The soil microbial community composition also changed with restoration years, with Actinobacteriota, Proteobacteria, Chloroflexi, and Acidobacteriota dominating the bacterial community, whereas Ascomycota and Basidiomycota dominating the fungal community. Fungal α-diversity generally increased with restoration years, whereas bacterial Shannon and Simpson indices exhibited unimodal patterns. Correlation analyses revealed strong associations among leaf functional traits, soil properties, and soil microbial communities. Partial least squares path modeling (PLS-PM) suggested that variations in soil properties and microbial communities were associated with changes in leaf functional traits, with soil properties showing the strongest association. Microbial communities exhibited weaker direct associations with leaf traits but may be indirectly linked to leaf trait variation through their relationships with soil conditions. These findings indicate that changes in leaf resource-use characteristics of *C. microphylla* during restoration years are closely associated with variation in soil conditions and microbial communities, highlighting the coordinated variation among plant, soil, and microbial components during the restoration of alpine sandy ecosystems restoration.

## 1. Introduction

Changes in precipitation patterns and the increasing frequency of extreme drought events driven by global climate change have profoundly affected the structure and functioning of arid and semi-arid ecosystems [1,2,3]. Arid and semi-arid regions are characterized by low precipitation, high evaporation, and nutrient-poor soils and are particularly susceptible to vegetation degradation and land desertification, making them among the most ecologically vulnerable regions worldwide [4,5]. Vegetation restoration is widely regarded as an important approach to improving regional ecological conditions. Vegetation restoration can progressively alter soil physicochemical conditions, and these changes are often accompanied by shifts in soil microbial community composition during ecosystem recovery [6]. As an important component of soil ecosystems, microbial communities play a critical role in ecosystem recovery through their involvement in organic matter decomposition, nutrient cycling, and plant nutrient acquisition [7]. However, under the combined constraints of prolonged drought and limited resource availability, the mechanism by which interactions among plants, soil, and microorganisms jointly regulate plant functional traits and shape ecological adaptation strategies during vegetation restoration remains poorly understood. This knowledge gap limits our understanding of the ecological processes and underlying mechanisms driving vegetation restoration in arid regions.

Plant leaf functional traits are key indicators of plant resource acquisition strategies and environmental adaptation, and are widely used to elucidate plant responses to environmental changes [8,9,10]. Specific leaf area (SLA) is closely associated with leaf construction costs, light capture capacity, and potential growth rate [11]. Species with higher SLA generally adopt a more resource-acquisitive strategy. Leaf dry matter content (LDMC) reflects the capacity of plants to retain nutrients and water and tolerate environmental disturbances [12], and species with higher LDMC tend to adopt a more conservative resource-use strategy. Plant adaptive strategies are not determined by a single trait but are jointly shaped by the coordination and trade-offs among multiple leaf functional traits [13,14]. Previous studies have shown that plant resource-use strategies can shift substantially during vegetation restoration or succession, often involving coordinated changes in leaf structural and nutrient-related traits along the resource acquisition-conservation spectrum [15,16]. However, existing studies on leaf functional traits have mainly focused on forest and grassland ecosystems, while patterns of variation in plant leaf functional traits and their ecological significance in alpine sandy ecosystems remain relatively poorly understood. Therefore, elucidating the variation and coordination of leaf functional traits during vegetation restoration in alpine sandy ecosystems is essential for improving our understanding of plant adaptation strategies under resource-limited conditions.

Soil microorganisms regulate soil nutrient availability through processes such as organic matter decomposition [7] and biological nitrogen fixation [17], thereby influencing plant growth and the expression of plant functional traits. Conversely, plants can modify soil microbial community composition and function through litter inputs [18], changes in the C:N ratio of litter [19], and the release of root exudates [20,21], thereby establishing dynamic feedbacks between plants and soil microbial communities. Previous studies have demonstrated close associations between plant functional traits and soil microbial communities. For example, higher leaf nitrogen content (LNC) is generally positively associated with bacteria-dominated microbial communities [22], whereas changes in leaf stoichiometric traits can significantly affect soil bacterial α-diversity [23]. However, during vegetation restoration, it remains unclear how shifts in soil conditions and microbial community structure are associated with changes in plant resource-use strategies and leaf functional traits. In this study, we focused on non-rhizosphere soil microbial communities because they are less directly influenced by short-term root effects [24] and may better reflect broader changes in the soil microbial environment associated with long-term vegetation restoration. Elucidating these linkages is important for understanding the coordination between aboveground plant functional adjustment and belowground ecological processes in alpine sandy ecosystems.

*Caragana microphylla* is a major shrub species widely used for vegetation restoration, windbreak establishment, and sand fixation in alpine sandy ecosystems. It is well adapted to cold and drought conditions. As a leguminous shrub, *C. microphylla* is capable of symbiotic nitrogen fixation, which represents an important ecological characteristic that may contribute to nutrient cycling and plant–soil interactions during vegetation restoration [25]. Previous studies have mainly focused on the soil physicochemical properties [26], plant community characteristics [27], and root traits [28] associated with *C. microphylla*. However, these studies have generally examined either aboveground vegetation characteristics or belowground soil processes separately, while the coordination between plant functional strategies and soil microbial communities during long-term restoration remains poorly understood. In particular, it remains unclear how leaf functional traits of *C. microphylla*, which reflect plant resource acquisition and conservation strategies, are associated with changes in soil properties and microbial community composition across different restoration stages in alpine sandy ecosystems. Therefore, an integrated understanding of plant–soil–microbial interactions is needed to elucidate the ecological responses of *C. microphylla* during vegetation restoration. Therefore, we investigated *C. microphylla* plantations with different restoration years (5, 10, 15, 23, and 36 Y) in an alpine sandy ecosystem. We systematically quantified leaf functional traits, soil properties, and soil microbial characteristics to elucidate patterns of variation in leaf functional traits during vegetation restoration and their associations with changes in soil microbial communities. Accordingly, we hypothesized that: (1) with increasing restoration years, leaf functional traits of *C. microphylla* exhibit coordinated variation and trade-offs reflecting shifts in plant resource-use strategies in relation to changes in soil conditions; and (2) variation in leaf functional traits of *C. microphylla* is associated with variation in soil properties and microbial communities along the restoration chronosequence, reflecting coordinated changes among plant traits, soil conditions, and microbial communities during vegetation restoration.

## 2. Results

### 2.1. Leaf Functional Traits of C. microphylla Along Restoration Years

**Figure 1:**
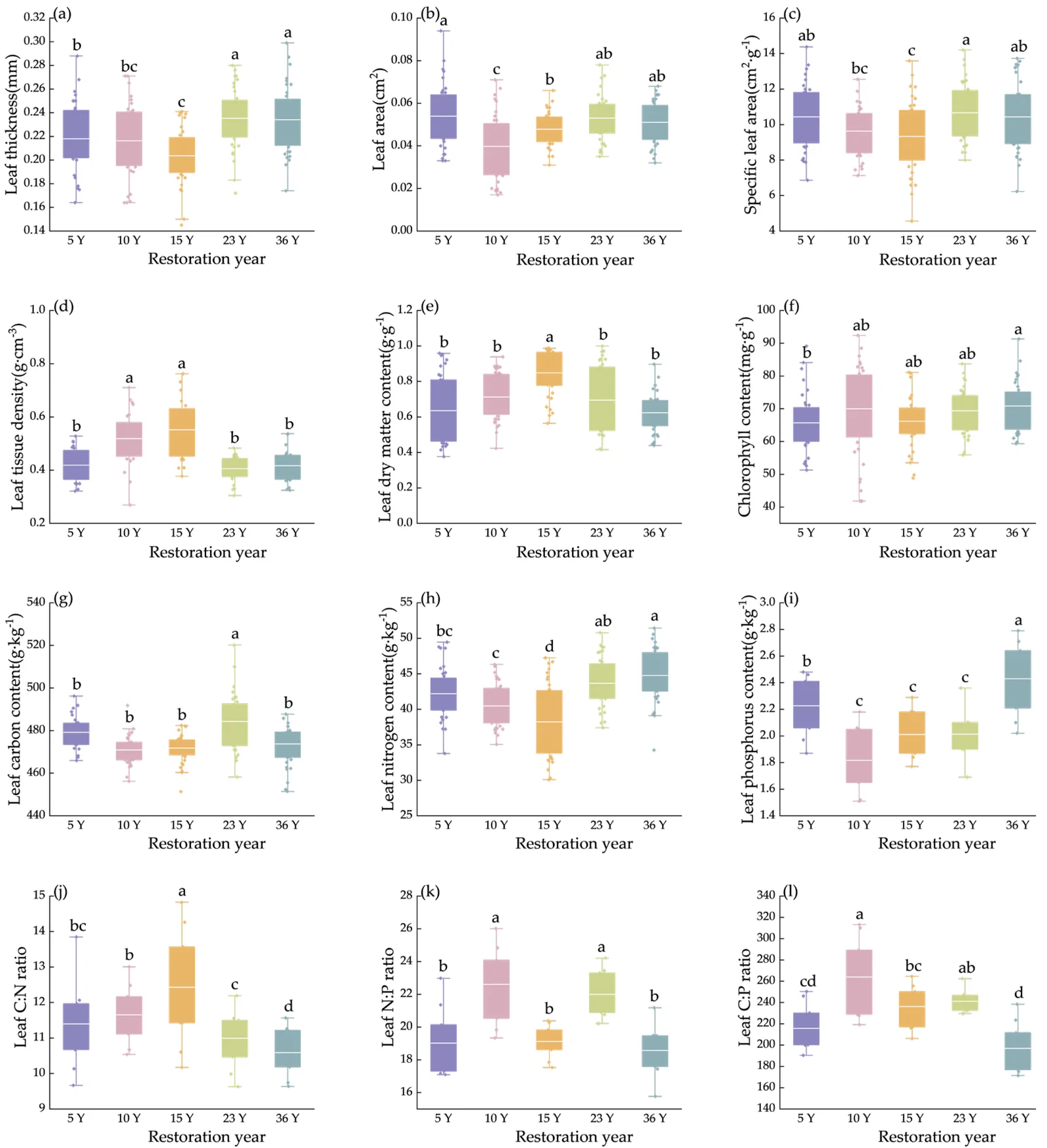
Leaf functional traits of *C. microphylla* plantations along different restoration years. (**a**) Leaf thickness (LT); (**b**) leaf area (LA); (**c**) specific leaf area (SLA); (**d**) leaf tissue density (LTD); (**e**) leaf dry matter content (LDMC); (**f**) chlorophyll content (Chl); (**g**) leaf carbon content (LCC); (**h**) leaf nitrogen content (LNC); (**i**) leaf phosphorus content (LPC); (**j**) leaf C:N ratio (LCNr); (**k**) leaf C:P ratio (LCPr); (**l**) leaf N:P ratio (LNPr). Different lowercase letters indicate significant differences among restoration years (*p* < 0.05).

With increasing vegetation restoration years, the leaf functional traits of *C*. *microphylla* differed significantly among restoration years (*p* < 0.05; Figure 1a–f), and exhibited distinct stage-dependent patterns along the restoration chronosequence. Specifically, leaf thickness (LT), leaf area (LA), and specific leaf area (SLA) showed a decreasing trend followed by an increasing trend with prolonged restoration years. In contrast, leaf tissue density (LTD) and leaf dry matter content (LDMC) exhibited the opposite pattern, initially increasing and then decreasing, with both reaching their maximum values at 15 Y, with values of 0.56 g·cm^−3^ and 0.85 g·g^−1^, respectively. Chlorophyll content (Chl) showed an overall increasing trend despite fluctuations among restoration stages, reaching its highest value of 70.82 mg·g^−1^ at 36 Y.

Leaf carbon, nitrogen, and phosphorus contents and their stoichiometric ratios also differed significantly among restoration years (*p* < 0.05, Figure 1g–i). Leaf carbon content (LCC) was significantly higher at 23 Y than at the other restoration years, whereas no significant differences were observed among 5 Y, 10 Y, 15 Y, and 36 Y (*p* > 0.05; Figure 1g). Leaf nitrogen content (LNC) and leaf phosphorus content (LPC) exhibited similar variation patterns of variation, both showing an initial decline followed by an increase with increasing restoration years. The LNC ranged from 38.23 to 44.77 g·kg^−1^ and reached its maximum value at 36 Y (Figure 1h). LPC ranged from 1.82 to 2.43 g·kg^−1^, and the values at 36 Y was significantly higher than those at all other restoration years (*p* < 0.05; Figure 1i). Compared with the 5 Y plantation, LPC increased by 9.11% at 36 Y, whereas no significant differences were observed among the other three restoration years (*p* > 0.05). The mean values of the leaf carbon-to-nitrogen ratio (LCNr), nitrogen-to-phosphorus ratio (LNPr), and carbon-to-phosphorus ratio (LCPr) ranged from 10.61 to 12.56, from 18.57 to 22.61, and from 196.90 to 263.91, respectively, with all three stoichiometric ratios reaching their minimum values at 36 Y (Figure 1j–l).

### 2.2. Responses of Soil Properties to Restoration Years

Electrical conductivity (EC), soil water content (SWC), soil organic carbon (SOC), soil total nitrogen (STN), and soil total phosphorus (STP) differed significantly among restoration years (*p* < 0.05, Table 1), whereas no significant differences were observed in pH or soil bulk density (SBD) (*p* > 0.05, Table 1). pH and SBD ranged from 7.56 to 7.96 and from 1.29 to 1.43 g·cm^−3^, respectively. EC showed a fluctuating pattern and reached its highest value at 10 Y (71.54 μS·cm^−1^). This value did not differ significantly from that observed at 36 Y but was significantly higher than those at the other restoration years. SWC was highest at 5 Y, decreased significantly thereafter, and increased again at 23 Y and 36 Y. SOC reached its highest value at 10 Y (2.12 g·kg^−1^), declined at 15 Y, and subsequently increased again. At 36 Y, SOC was approximately 103% higher than that at 5 Y. Soil inorganic carbon (SIC) content was highest at 5 Y (11.55 g·kg^−1^), and was significantly higher than at the other restoration years (*p* < 0.05). STN showed an overall increasing trend with restoration years, increasing from 0.10 g·kg^−1^ at 5 Y to 0.23 g·kg^−1^ at 36 Y. In contrast, STP reached its highest value at 10 Y (0.53 g·kg^−1^) and subsequently showed an overall decreasing trend, declined to 0.34 g·kg^−1^ at 36 Y.

### 2.3. Responses of Soil Microbial Community Structure to Restoration Years

**Figure 2:**
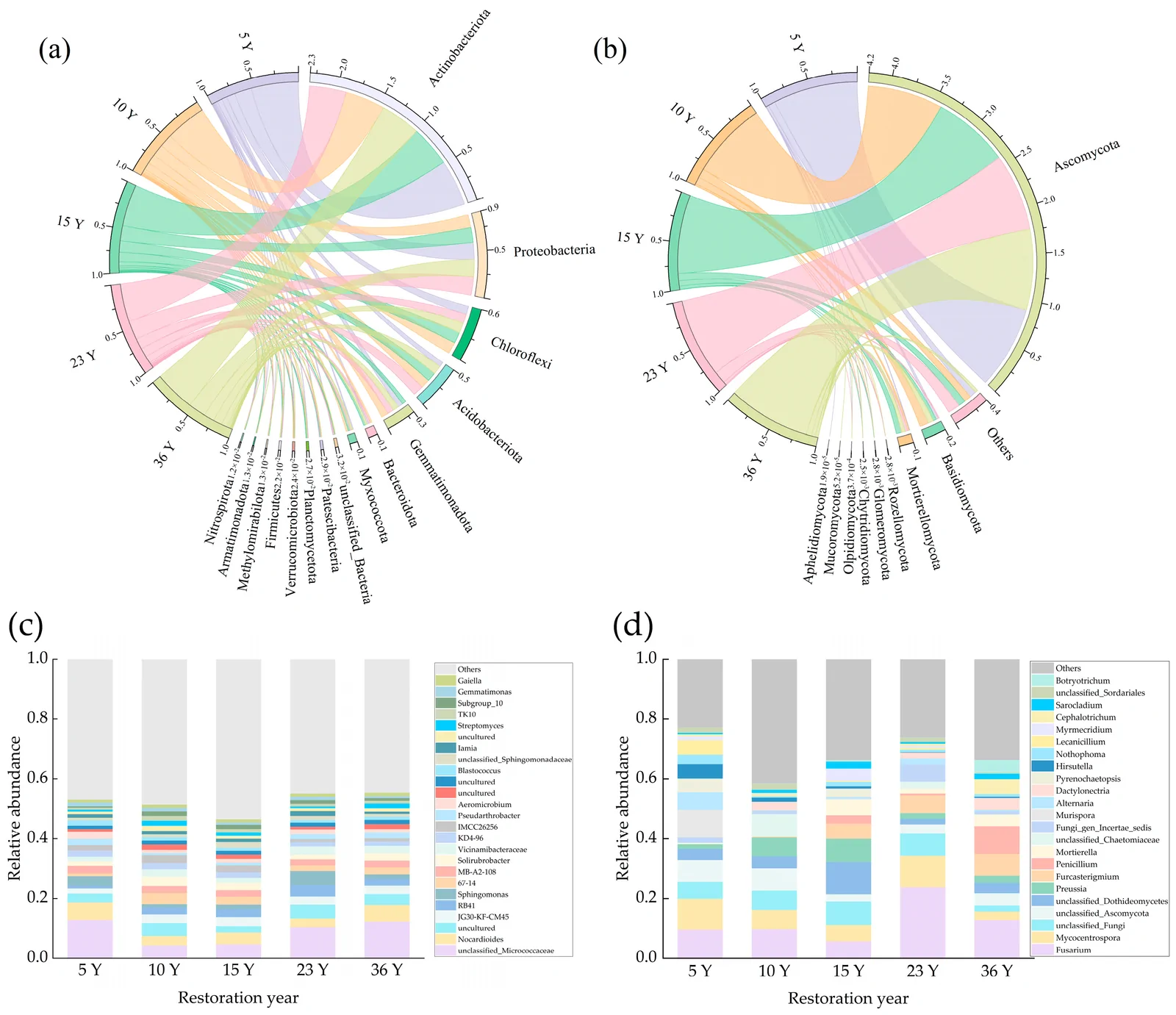
Soil bacterial and fungal community compositions along different restoration years. (**a**) Bacterial community composition at the phylum level; (**b**) fungal community composition at the phylum level; (**c**) bacterial community composition at the genus level; (**d**) fungal community composition at the genus level.

The composition of soil microbial communities in *C. microphylla* plantations varied among restoration years (Figure 2). At the phylum level, 15 bacterial phylum had relative abundances greater than 1% across all soil samples. Actinobacteriota, Proteobacteria, Chloroflexi, and Acidobacteriota were the dominant bacterial phylum, with mean relative abundances of 46.36%, 18.29%, 11.45%, and 9.63%, respectively, together accounting for more than 85% of the bacterial community (Figure 2a). The relative abundances of Actinobacteriota and Proteobacteria initially decreased and subsequently increased with restoration years, reaching their minimum values at 23 Y and 10 Y, respectively. In contrast, Chloroflexi and Acidobacteriota showed opposite patterns, generally increasing initially and then decreasing, with their maximum relative abundances observed at 10 Y and 23 Y, respectively. At the genus level, 25 dominant taxa with relative abundance >1% were included in the analysis (Figure 2c). Unclassified_Micrococcaceae, *Nocardioides*, *Sphingomonas*, *Solirubrobacter*, *Pseudarthrobacter*, *Streptomyces*, and *Gaiella* were among the dominant taxa. Unclassified_Micrococcaceae showed relatively high abundance at 5 Y, 23 Y, and 36 Y, whereas *Nocardioides* was more abundant at 5 Y and 36 Y. *Sphingomonas* showed a fluctuating pattern and reached its highest relative abundance at 23 Y (4.71%). Overall, the relative abundances of dominant bacterial taxa varied among restoration stages.

Ascomycota and Basidiomycota were the dominant fungal phylum, with mean relative abundances of 84.71% and 4.44%, respectively, together accounting for 89.15% of the fungal community (Figure 2b). The relative abundance of Ascomycota initially decreased and subsequently increased with restoration years, reaching a minimum value of 81.26% at 10 Y. In contrast, the relative abundance of Basidiomycota generally increased initially and then decreased, reaching a maximum value of 9.37% at 10 Y. At the genus level, 23 dominant taxa with relative abundance >1% were included in the analysis (Figure 2d). The dominant classified genera mainly included *Fusarium*, *Mycocentrospora*, *Preussia*, *Furcasterigmium*, *Penicillium*, *Mortierella*, *Murispora*, *Alternaria*, and *Hirsutella*. *Fusarium* showed marked variation among restoration stages and reached its highest relative abundance at 23 Y (23.84%). *Mycocentrospora* was relatively abundant at 5 Y and 23 Y, whereas *Preussia* peaked at 15 Y. *Furcasterigmium* and *Penicillium* showed their highest relative abundances at 36 Y. These results indicated clear stage-dependent variation in the relative abundances of dominant fungal taxa along the restoration chronosequence.

### 2.4. Responses of Soil Microbial α-Diversity to Different Restoration Years

**Figure 3:**
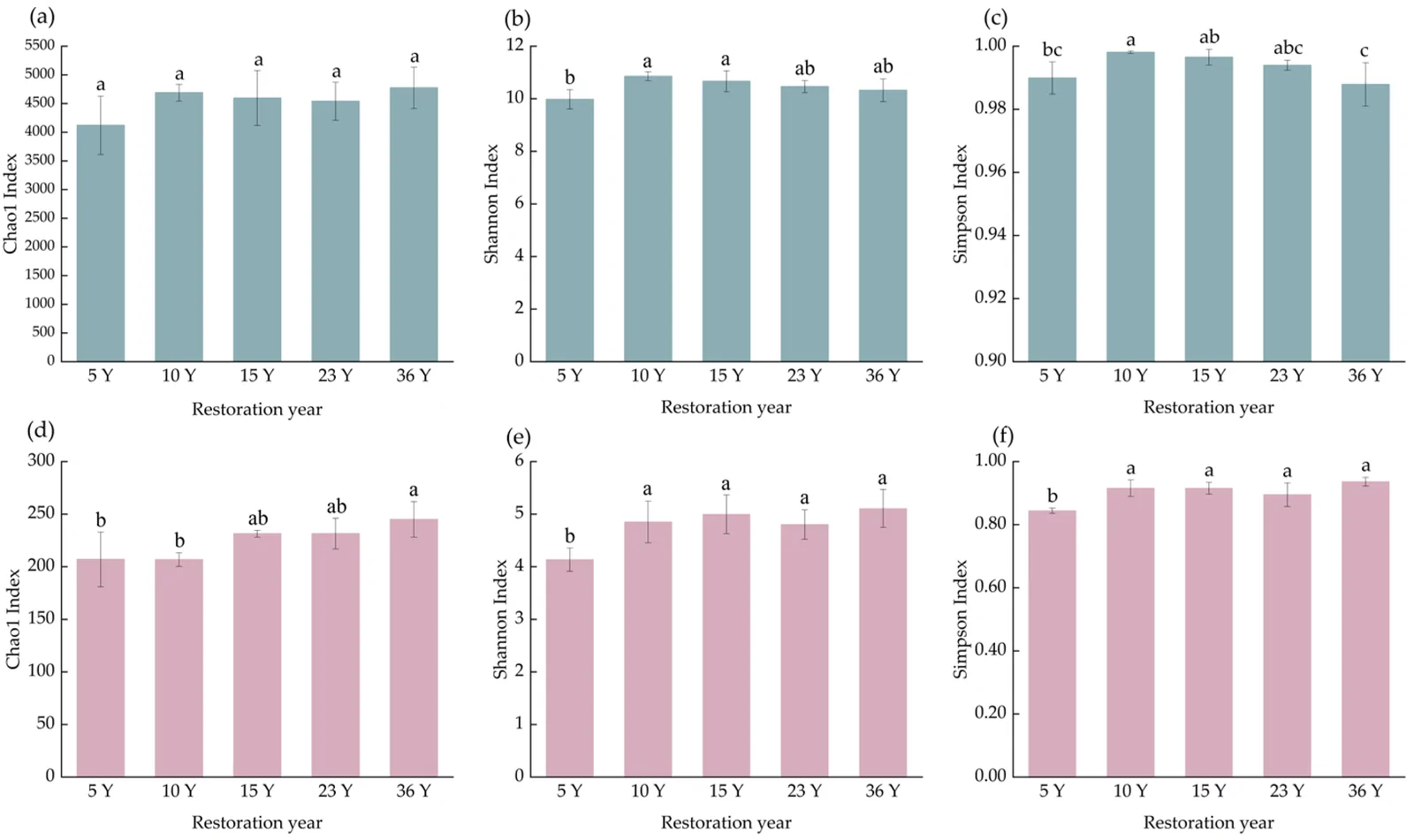
Analysis of α-diversity of soil microbial communities along different restoration years. (**a**–**c**) Chao1, Shannon, and Simpson indices of soil bacterial communities, respectively; (**d**–**f**) Chao1, Shannon, and Simpson indices of soil fungal communities, respectively. Different lowercase letters indicate significant differences among restoration years (*p* < 0.05).

The α-diversity of soil bacterial and fungal communities varied among different restoration years in *C. microphylla* plantations (Figure 3a–f). For bacterial communities, the Shannon and Simpson indices showed similar patterns, initially increasing and then decreasing with restoration years, with both reaching their maximum values at 10 Y. In contrast, the bacterial Chao1 index showed no significant differences among restoration years (*p* > 0.05). For fungal communities, the Chao1, Shannon, and Simpson indices showed an overall increasing tendency with restoration years and reached their maximum values at 36 Y, although slight fluctuations were observed among the intermediate restoration stages. Moreover, the fungal Shannon and Simpson indices at 5 Y were significantly lower than those at the other restoration years (*p* < 0.05).

### 2.5. Correlations Among Leaf Functional Traits, Soil Properties and Microbial Communities

**Figure 4:**
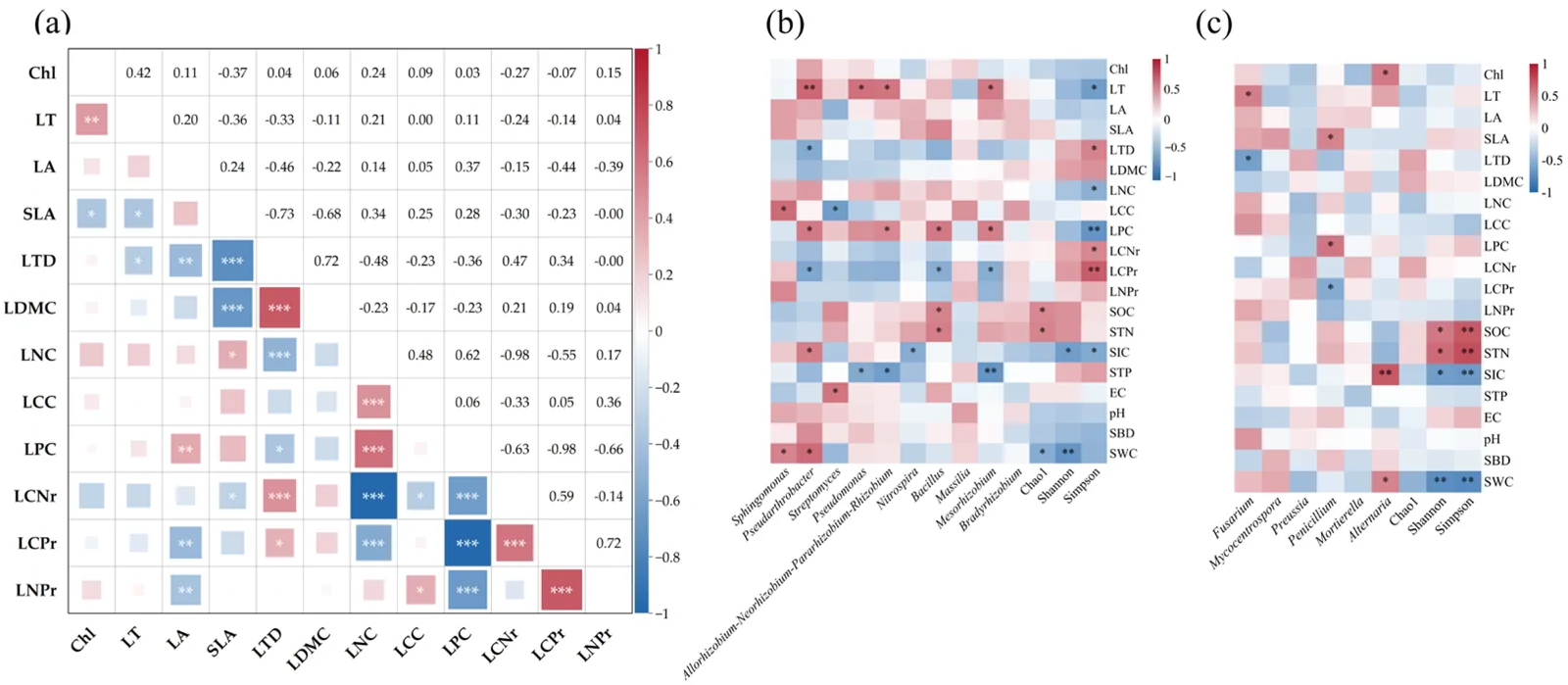
Correlations among leaf functional traits, soil microbial communities, and soil properties. (**a**) Correlations among leaf functional traits; (**b**) bacterial genera and α-diversity indices; (**c**) fungal genera and α-diversity indices. * *p* < 0.05, ** *p* < 0.01, and *** *p* < 0.001.

Pearson correlation analysis was performed on 12 leaf functional traits of *C. microphylla* across the restoration years. The results showed widespread significant correlations and trade-off relationships among the traits (Figure 4a). LT was negatively correlated with SLA and LTD (*p* < 0.05), and significantly positively correlated with Chl (*p* < 0.01). LA was negatively correlated with LTD, LCPr, and LNPr, and significantly positively correlated with LPC (*p* < 0.01). SLA was negatively correlated with LTD and LDMC (*p* < 0.001). LTD was positively correlated with LDMC and LCNr, but negatively correlated with LNC (*p* < 0.001). LPC was negatively correlated with LCNr, LCPr, and LNPr (*p* < 0.001).

At the bacterial genus level, several dominant taxa were significantly associated with leaf traits and soil properties (Figure 4b). *Mesorhizobium* and *Bacillus* showed positive correlations with LPC, whereas both taxa were negatively correlated with LCPr (*p* < 0.05). *Bacillus* showed positive correlations with SOC and STN (*p* < 0.05). *Pseudarthrobacter* was negatively associated with LTD and LCPr, while showing significant correlations with LPC, SIC, and SWC (*p* < 0.05). *Sphingomonas* was positively correlated with LCC and SWC, whereas *Streptomyces* showed a negative correlation with LCC and a positive correlation with EC (*p* < 0.05). Bacterial diversity indices showed significant associations with soil properties and leaf traits. Chao1 was positively correlated with SOC and STN but negatively correlated with SWC (*p* < 0.05). Shannon was negatively correlated with SIC and SWC, whereas Simpson showed significant associations with LNC, LPC, LCNr, LCPr, and SIC (*p* < 0.05).

At the fungal genus level, several dominant taxa showed significant associations with leaf traits and soil properties (Figure 4c). *Fusarium* showed a negative correlation with LTD but a positive correlation with LT (*p* < 0.05). *Penicillium* was positively correlated with SLA and LPC but negatively correlated with LCPr (*p* < 0.05). *Alternaria* showed positive correlations with SIC, SWC, and Chl (*p* < 0.05). Furthermore, fungal Shannon and Simpson indices exhibited similar correlation patterns, being positively correlated with SOC and STN but negatively correlated with SIC and SWC (*p* < 0.05).

### 2.6. Relationships of Leaf Functional Traits with Soil Microbial Communities and Soil Properties

**Figure 5:**
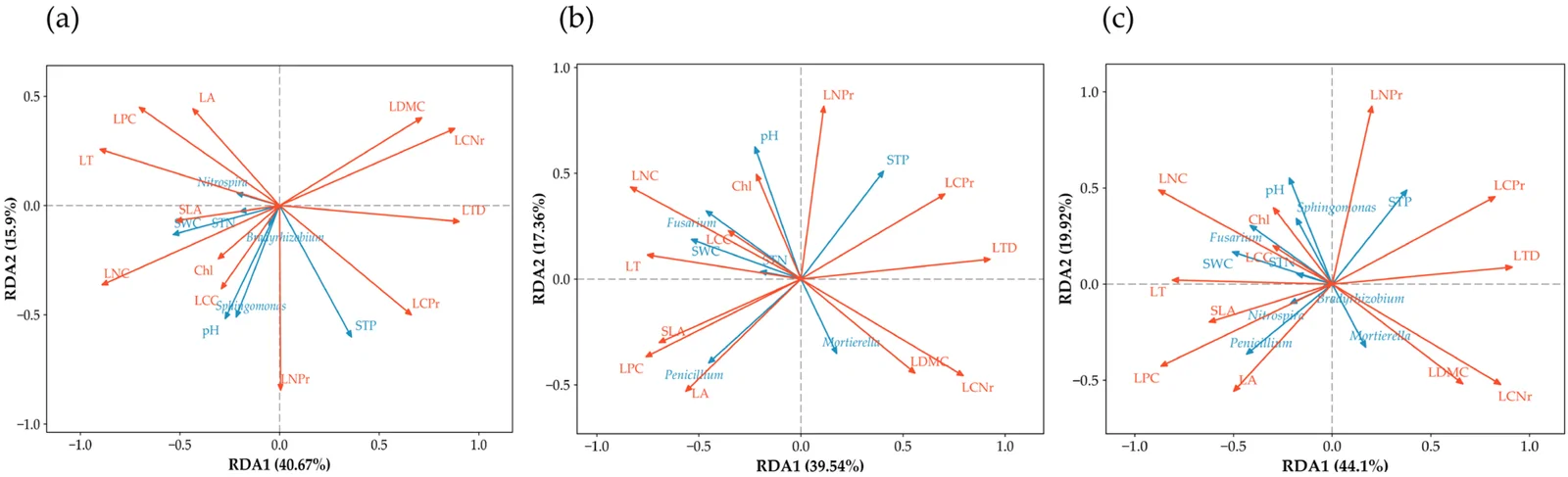
RDA plots illustrating the associations between leaf functional traits and explanatory variables including soil properties and dominant microbial genera. (**a**) Bacterial genera combined with soil properties; (**b**) fungal genera combined with soil properties; (**c**) both bacterial and fungal genera together with soil properties. Red arrows represent response variables (leaf functional traits), whereas blue arrows denote explanatory variables.

Redundancy analysis (RDA), with soil bacterial community composition and soil properties as explanatory variables and leaf functional traits as response variables, showed that the first and second ordination axes explained 40.67% and 15.90% of the variation in leaf functional traits, respectively, accounting for 56.57% of the total variation (Figure 5a). The overall RDA model was significant based on the Monte Carlo permutation test (F = 2.89, *p* = 0.002). The vector for *Nitrospira* was oriented in a similar direction to those for LA and LPC, but in the opposite direction to those for LTD and LCPr. SLA was closely aligned with STN, while LNC and SWC showed similar orientations. The vectors for *Sphingomonas*, LCC, and Chl were oriented in similar directions, and pH was also oriented toward Chl. STP showed a similar direction to LCPr and LNPr.

The RDA of the relationships among soil fungal community composition, soil properties, and leaf functional traits showed that the first two ordination axes explained 39.54% and 17.36% of the variation in leaf functional traits, respectively, accounting for a cumulative 56.90% of the variation (Figure 5b). The overall RDA model was significant based on the Monte Carlo permutation test (F = 2.28, *p* = 0.008). The vector for *Fusarium* was closely aligned with those for LCC and LNC, and SWC was oriented in a similar direction. *Penicillium* was oriented in a similar direction to SLA and LPC, while *Mortierella* pointed in the opposite direction to LNPr.

When soil bacterial and fungal community composition and soil properties were jointly included as explanatory variables, the first and second RDA axes explained 44.10% and 19.92% of the variation in leaf functional traits, respectively, accounting for 64.02% of the total variation (Figure 5c). The overall model was significant based on the Monte Carlo permutation test (F = 3.27, *p* = 0.001). *Penicillium* and *Nitrospira* were oriented in similar directions to SLA and LPC, respectively. *Mortierella* was oriented in a similar direction to LDMC and LCNr. *Fusarium* and *Sphingomonas* showed a similar orientation to Chl, LCC, LNC and pH, whereas STP was oriented similarly to LNPr and LCPr.

**Figure 6:**
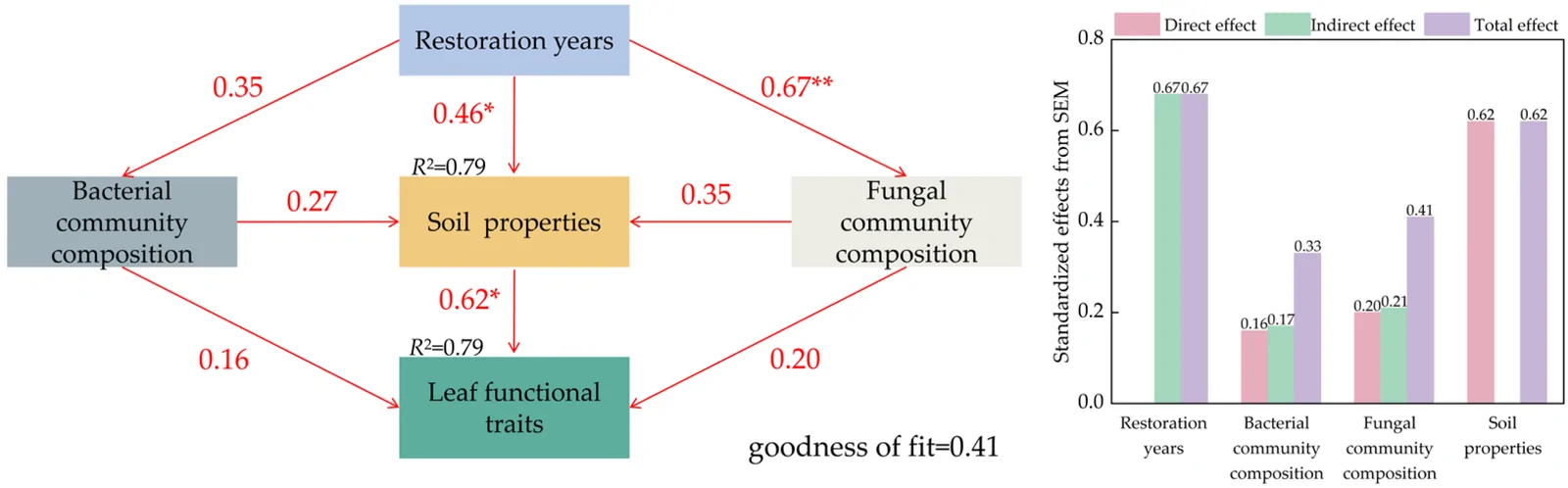
Partial least squares path modeling (PLS-PM) showing the associations of restoration years, soil properties, and soil microbial communities on leaf functional traits. Red arrows indicate positive effects, respectively. The values associated with arrows are standardized path coefficients. Asterisks indicate the significance of two-sided *t*-tests based on the PLS-PM inner-model regressions: * *p* < 0.05, ** *p* < 0.01.

Partial least squares path modeling (PLS-PM) was used to characterize the statistical associations among restoration years, soil properties, microbial community composition, and leaf functional traits (Figure 6). The model goodness of fit value was 0.41, indicating that the model provides an acceptable fit to the data. The model showed that restoration years were indirectly associated with leaf functional traits through changes in soil properties and microbial community composition. Restoration years exhibited positive associations with soil properties (0.46), bacterial community composition (0.35), and fungal community composition (0.67). Soil properties showed the strongest direct association with leaf functional traits (0.62). Both bacterial and fungal communities were associated with leaf functional traits, with direct path coefficients of 0.16 and 0.20, respectively. The model also showed that bacterial and fungal community compositions were indirectly associated with leaf functional traits via soil properties.

## 3. Discussion

### 3.1. Changes in Leaf Functional Traits and Adaptive Strategies of C. microphylla Plantations Across Restoration Years

Plant functional traits can reflect ecological strategies related to resource acquisition, resource use, and stress tolerance, and thus provide an important basis for understanding plant adaptation to environmental change [29,30]. Our results support the first hypothesis, showing that the leaf functional traits of *C. microphylla* in alpine sandy land ecosystem exhibited significant stage-dependent changes along restoration years rather than simple linear patterns. LA, LT, and SLA initially decreased and subsequently increased, whereas LTD and LDMC showed the opposite trend, initially increasing and then decreasing, with relatively high values observed at 15 Y. These coordinated changes are consistent with stage-dependent adjustments in leaf structural investment and resource-use strategies along the restoration chronosequence.

During the early-to-middle stages of restoration, particularly at 15 Y, *C. microphylla* exhibited a relatively conservative combination of leaf traits, characterized by lower SLA and higher LTD and LDMC. Higher LTD and LDMC generally indicate denser leaf tissues and greater investment in structural materials, which may help reduce the risk of water loss and enhance leaf tolerance to drought, low temperature, and nutrient limitation [31]. During the later stage of restoration (15–36 Y), however, these trends were reversed: SLA increased, whereas LTD and LDMC decreased toward 36 Y, accompanied by increases in LNC and LPC, a trait combination associated with enhanced carbon assimilation and resource-use efficiency [11,32,33]. This combination of traits is consistent with a relatively more acquisitive resource-use strategy. Therefore, our results suggest that leaf resource-use strategies exhibited non-linear, stage-dependent shifts during vegetation restoration, with an initial transition toward greater resource conservation from 5 to 15 Y, followed by a shift toward a relatively more acquisitive strategy from 15 to 36 Y.

Numerous studies have shown that coordination and trade-offs among leaf functional traits represent important mechanisms by which plants optimize resource use and adapt to environmental variation [34,35]. Correlation analysis showed that LT was negatively correlated with SLA, consistent with previous studies [36,37]. This relationship suggests that greater leaf thickness may restrict heat exchange between leaves and the atmosphere and reduce the diffusion of water vapor and CO_2_ into and out of leaves [38,39]. In addition, SLA was significantly and negatively correlated with LDMC, which is consistent with the global leaf economics spectrum and the findings of previous studies [40,41]. This indicates that the expression and coordination of leaf functional traits in *C. microphylla* are broadly consistent with those observed in other plant species, whereby plants adapt to water and nutrient limitation by coordinating leaf construction costs and tissue density [41,42,43].

We also found a highly significant negative correlation between LNPr and LPC, whereas LNPr was not significantly correlated with LNC, suggesting a potentially greater role of phosphorus than nitrogen in constraining plant growth. This finding is consistent with the results reported by Song et al. (2022) for leaf functional traits across different ages of *Zanthoxylum planispinum* [44]. In the present study, the mean LNPr values of *C. microphylla* ranged from 18.57 to 22.61 across restoration years. According to the N:P threshold proposed by Koerselman and Meuleman [45], an N:P > 16 is generally considered indicative of possible P limitation rather than conclusive evidence of P limitation. This suggests that phosphorus availability may play a relatively important role in regulating the nutrient balance of *C. microphylla* during restoration. However, direct evidence of phosphorus limitation would require measurements of available phosphorus and other indicators of nutrient status.

Overall, *C. microphylla* exhibited non-linear and stage-dependent changes in leaf resource-use strategies during restoration. The conservative trait combination was most evident at 15 Y, followed by a shift toward relatively more acquisitive traits at later restoration stages. These results suggest that leaf functional strategies changed dynamically along the restoration chronosequence rather than following a simple directional transition.

### 3.2. Responses of Soil Microbial Communities to Restoration Years

Vegetation restoration can alter soil microbial community composition and diversity by modifying vegetation coverage, litter inputs, root activity, and soil resource availability [46]. In this study, the dominant bacterial phylum in the soil of *C. microphylla* artificial shrub plantations across different restoration years were Actinobacteriota, Proteobacteria, Chloroflexi, and Acidobacteriota, while the dominant fungal phylum were primarily Ascomycota and Basidiomycota. These patterns are consistent with those reported in previous studies of soil microbial communities along vegetation restoration chronosequences [47,48].

The composition of soil bacterial communities exhibited distinct successional patterns along the restoration chronosequence. Along the restoration chronosequence, the relative abundance of Actinobacteriota initially decreased and subsequently increased, reaching a minimum of 41.92%; however, it remained the dominant bacterial phylum throughout the restoration process. The relative abundance of Proteobacteria remained relatively stable (14.52–19.10%) across restoration stages. The relatively stable abundance of this phylum may reflect its broad ecological adaptability and functional diversity. Previous studies have shown that Proteobacteria comprise diverse taxa with various metabolic capabilities and are involved in soil biogeochemical processes, including organic matter decomposition and carbon and nitrogen cycling [49]. At the genus level, the relative abundance of dominant genera in the soil of *C*. *microphylla* stands varied among different restoration years. The relative abundance of *Sphingomonas* reached its highest value at 23 Y (4.71%). Previous studies have shown that some *Sphingomonas* taxa can form beneficial interactions with plants and participate in carbon cycling [50]; therefore, the observed variation in their relative abundance may be associated with plant–microbe interactions. With respect to fungal communities, Ascomycota was the overwhelmingly dominant phylum, consistent with previous findings for *Hippophae rhamnoides* plantations in sandy ecosystems [51]. The strong environmental adaptability of Ascomycota may be attributed to its ability to produce resistant spores and develop relatively robust cell walls, enabling it to withstand environmental stresses [52]. However, the dominance of Ascomycota should be interpreted with caution, as amplicon sequencing characterizes the relative composition of fungal communities rather than absolute fungal biomass or activity. Therefore, the high relative abundance of Ascomycota observed in this study indicates its prevalence within the detected fungal community, but does not necessarily reflect its absolute biomass or a greater ecological contribution than other fungal taxa.

In this study, soil bacterial α-diversity in *C. microphylla* plantations exhibited a unimodal pattern along the restoration chronosequence, initially increasing and subsequently decreasing as the number of restoration years increased. This pattern was consistent with the responses of soil microbial communities observed during vegetation restoration in the Horqin Sandy Land reported by Yao et al. (2026) [48]. In contrast, fungal α-diversity showed an overall increasing trend with restoration years, which may be associated with the continuous accumulation of organic matter during vegetation recovery. Increased organic matter availability provides more diverse carbon substrates for fungal colonization, growth, and organic matter decomposition [53]. These results indicate that soil microbial community composition and α-diversity vary substantially along the restoration chronosequence, while bacterial and fungal communities exhibit distinct patterns of variation with restoration years.

### 3.3. Responses of Leaf Functional Traits of C. microphylla to Soil Properties and Microbial Communities

Plant leaf functional traits are commonly associated with a trade-off between resource acquisition and conservation strategies, and their variation may be related to soil water conditions, nutrient status, and belowground biological processes [22]. Redundancy analysis (RDA) showed that variation in the leaf functional traits of *C. microphylla* was closely associated with soil properties and soil microbial community composition. This result is consistent with our second hypothesis that changes in soil properties and microbial communities are closely associated with variation in leaf functional traits during vegetation restoration. Distinct microbial taxa exhibited different associations with leaf functional traits, suggesting that variation in belowground microbial community composition was closely related to variation in plant resource-use characteristics. In the bacterial community, the relative abundances of *Sphingomonas* and *Bradyrhizobium* were positively associated with resource-acquisitive leaf traits, such as LNC, LCC and SLA. *Sphingomonas* and *Bradyrhizobium* both belong to the phylum Proteobacteria, whereas *Nitrospira* is affiliated with the phylum Nitrospirota. Previous studies have shown that some Proteobacteria taxa exhibit relatively high metabolic activity and rapid growth responses, and are considered to be relatively more abundant in environments with sufficient organic carbon and nutrient availability [48]. Fungal communities also exhibited significant coordinated variation with leaf functional traits. RDA showed that the relative abundances of *Fusarium* were closely associated with leaf nutrient-related traits (LNC and LPC) and soil properties (SWC and STN). *Fusarium* belonging to the phylum Ascomycota. Previous studies have shown that some Ascomycota taxa possess the capacity to decompose complex organic compounds, including cellulose and lignin [54,55]. Therefore, the observed associations among these fungal taxa, soil nutrient condition, and leaf nutrient traits may reflect their potential involvement in organic matter transformation and nutrient cycling processes. However, as these processes were not directly measured, this explanation remains a literature-based ecological interpretation rather than a functionally proven mechanism.

Partial least squares path modeling (PLS-PM) further characterized the direct and indirect statistical associations among restoration years, soil properties, and soil microbial communities, and leaf functional traits. The model showed that restoration years were associated with variation in soil properties and microbial community composition, particularly fungal communities. Among the examined factors, soil properties exhibited the strongest direct association with leaf functional traits, suggesting that variations in soil conditions were closely linked to changes in leaf functional traits along the restoration chronosequence. Although both bacterial and fungal communities showed direct associations with leaf functional traits, their path coefficients were relatively samll, indicating that microbial community composition may have a comparatively limited direct association with leaf trait variation. In addition, microbial communities may be indirectly associated with leaf functional traits through their relationships with soil properties. Given that this study was based on a chronosequence approach, the PLS-PM results should be interpreted as statistical associations rather than evidence of direct ecological causality.

Overall, soil properties were closely associated with variation in both plant leaf functional traits and soil microbial communities, highlighting the coordinated relationships among plant, soil, and microbial components during vegetation restoration in alpine sandy ecosystems. Variation in leaf functional traits of *C. microphylla* along the restortion chronosequence was closely associated with concurrent changes in soil conditions and microbial community structure. By integrating leaf traits, soil properties, and both bacterial and fungal communities, this study provides a comprehensive view of their coordinated variation across the restoration chronosequence. Future studies incorporating comparisons between rhizosphere and bulk soils, analyses of microbial functional genes, and manipulative experiments such as nutrient addition are needed to clarify the mechanisms underlying plant–soil–microbe relationships during ecosystem restoration.

## 4. Materials and Methods

### 4.1. Study Area

The study area is located at the Qinghai Gonghe Desert Ecosystem Research Station (36°03′–36°40′ N, 99°45′–100°30′ E). Elevation ranges between 2871 and 3870 m within our study area [56]. The climate is characterized by alpine arid conditions, with a mean annual temperature is 2.4 °C and mean annual precipitation is 246.3 mm. Precipitation occurs primarily from July to September. Mean potential evaporation is 1716.7 mm, and the mean annual frost-free period is approximately 91 days [57]. According to the USDA Soil Taxonomy, the predominant soils in the study area are classified as Psamments, with the fine sand fraction (100–250 μm) constituting the dominant particle-size class. Artificially established vegetation is dominated by shrub species such as *C*. *microphylla*, *Salix cheilophila*, and *Tamarix chinensis*, whereas the natural vegetation mainly comprises *Artemisia desertorum*, *Stipa breviflora*, and *Neotrinia splendens*. Since the initiation of artificial vegetation restoration at the research station in 1958 [58], a combination of mechanical and biological sand-stabilization measures have been implemented, including the installation of checkerboard sand barriers and the planting of psammophytic species. These restoration practices have contributed to vegetation establishment and sand stabilization in the study area. The selected restoration sites were located within the same desert ecosystem and were subjected to comparable restoration practices, with similar topographic conditions, soil backgrounds, and management histories. Therefore, restoration year was used as the primary chronosequence gradient for examining vegetation restoration patterns in this study.

### 4.2. Field Investigation

The artificial afforestation years of *C. microphylla* in the study area are well documented, providing a relatively complete vegetation restoration chronosequence. In this study, *C. microphylla* artificial shrublands representing five restoration years (5, 10, 15, 23, and 36 Y) were selected to investigate variation in leaf functional traits, soil microbial characteristics, and their relationships across different restoration stages. Three replicate plots within each restoration year were spatially separated by 200–300 m. Within each plot, one 20 m × 20 m shrub quadrat was established, and three 1 m × 1 m herbaceous quadrats were randomly placed within each shrub quadrat. Within each shrub quadrat, shrub community characteristics, including the number of individuals, plant height, basal diameter, coverage, and frequency, were recorded. Within the herbaceous quadrats, the number of tussocks, plant height, coverage, and frequency were measured. These measurements were used to characterize vegetation community structure across different restoration stages. Detailed information on the sampling sites is provided in Table 2.

### 4.3. Sample Collection

Field sampling was conducted in August 2024. For each restoration year, three spatially independent plots were selected as biological replicates (n = 3). In each plot, 12 healthy and similarly developed *C. microphylla* individuals were randomly selected for leaf sampling. Fully expanded, undamaged mature leaves were collected from the outer canopy in each of the four cardinal directions of east, south, west, and north directions of each individual. Ten leaves per plant were randomly selected for measurement of LT and LA, while additional leaf samples were collected for the determination of carbon, nitrogen, and phosphorus contents. All samples were labeled according to individual plants, placed in sealed polyethylene bags, and transported to the laboratory for further analyses. In addition, three healthy *C. microphylla* individuals within each plot were randomly selected for soil sampling. Surface soil samples (0–20 cm) were collected using a soil auger at a distance of 50–70 cm from the base of each plant. After removing plant roots, stones, and other debris, the soil samples were combined in equal proportions by mass, thoroughly homogenized, and divided into two portions. One portion was air-dried and sieved for subsequent physicochemical analyses. The other portion of fresh soil was immediately stored at −80 °C for subsequent analysis of soil microbial community analysis. In addition, three undisturbed soil cores were collected separately from the 0–20 cm soil layer in each plot using a 100 cm^3^ sampling ring for subsequent determination of soil bulk density (SBD) and soil water content (SWC).

### 4.4. Measurement of Variables

#### 4.4.1. Measurement of Leaf Functional Traits

Leaf thickness (LT) was measured using a vernier caliper with a precision of 0.01 mm. Because individual leaflets of *C*. *microphylla* were small and difficult to measure accurately, 10 leaflets were aligned in the same orientation and stacked together. The cumulative thickness of the 10 leaflets was measured at the basal, middle, and apical portions along their longitudinal axes of the leaflets, avoiding the main veins as much as possible. The mean of the three measurements was taken as the cumulative thickness of the 10-leaflet stack. This procedure was repeated for multiple sets of leaflets, and the mean cumulative thickness was divided by the number of leaflets to obtain the thickness of a single leaflet. After surface impurities and petioles were removed, leaf area (LA, cm^2^) was determined by scanning the leaves with an image analysis system (WinRHIZO, Regent Instruments Inc., Quebec City, QC, Canada), while leaf length (LL, cm) and leaf width (LW, cm) were determined from the scanned images using the pixel-based measurements method in Adobe Photoshop 2024. Fresh leaf weight (LFW, g) was measured using an electronic balance (precision: ±0.001 g). The leaves were then placed in envelopes, oven-dried at 105 °C for 30 min for enzyme inactivation, and subsequently oven-dried at 75 °C to a constant weight to determine leaf dry weight (LDW, g). Leaf dry matter content (LDMC, g·g^−1^) and specific leaf area (SLA, cm^2^·g^−1^) were calculated using the following equations: LDMC = LDW/LFW and SLA = LA/LDW. A subsample of oven-dried *C. microphylla* leaves was ground using a ball mill and passed through a 100-mesh sieve. Leaf carbon content (LCC, g·kg^−1^) and leaf nitrogen content (LNC, g·kg^−1^) were determined using an EA3000 CHNS-O elemental analyzer (EuroVector S.p.A., Pavia, Italy). For phosphorus analysis, a portion of the leaf powder was digested with H_2_SO_4_-H_2_O_2_. Leaf phosphorus content (LPC, g·kg^−1^) was then determined in the digest using the molybdenum–antimony–ascorbic acid colorimetric method with a spectrophotometer (UV-1900i, Shimadzu Corporation, Kyoto, Japan).

#### 4.4.2. Measurement of Soil Properties

The measured soil properties included soil pH, electrical conductivity (EC, μS·cm^−1^), SBD (g·cm^−3^), SWC (%), and the contents of soil organic carbon (SOC), soil total nitrogen (STN), and soil total phosphorus (STP) (g·kg^−1^). Soil pH and EC were measured in soil-deionized water suspensions at soil-to-water ratios of 1:2.5 and 1:5 (*w*/*v*), respectively, using a pH meter (pHS-25, INESA Instrument, Shanghai, China) and a conductivity meter (Model AZ-8360, AZ Instrument Corp., Taichung, Taiwan, China), respectively. SBD was determined using the core method. SBD was calculated as the oven-dry mass of the soil sample divided by the volume of the core sampler (100 cm^3^). SWC was determined gravimetrically. Immediately after sampling, the fresh soil mass was recorded, after which the samples were oven-dried at 105 °C to constant mass and weighed again. SWC was calculated as the difference between the fresh and oven-dried soil masses divided by the oven-dried soil mass and was expressed as a percentage. Soil total carbon (STC) and STN contents were determined using an elemental analyzer (FlashSMART, Thermo Fisher Scientific, Waltham, MA, USA). SOC was determined using the potassium dichromate-concentrated sulfuric acid oxidation method with external heating. Briefly, soil samples were oxidized with K_2_Cr_2_O_7_ and concentrated H_2_SO_4_ at approximately 170–180 °C for 5 min, and the residual dichromate was quantified by titration with FeSO_4_. No separate acid pretreatment was applied to remove inorganic carbon prior to SOC determination because SOC was quantified using the dichromate oxidation method. Soil inorganic carbon (SIC) was subsequently calculated as SIC = STC-SOC. Soil total phosphorus (STP) was determined following HNO_3_-HClO_4_-HF digestion, followed by inductively coupled plasma optical emission spectrometry (ICP-OES).

#### 4.4.3. Sequencing of Soil Microbial Diversity

Total DNA was extracted from approximately 0.5 g of homogenized fresh soil using the MagBeads FastDNA Kit for Soil (116564384) (MP Biomedicals, Santa Ana, CA, USA). The V3-V4 region of the bacterial 16S rRNA was amplified using primers 338F (5′-ACTCCTACGGGAGGCAGCA-3′) and 806R (5′-GGACTACHVGGGTWTCTAAT-3′). The fungal ITS1 was amplified using primers ITS5 (5′-GGAAGTAAAAGTCGTAACAAGG-3′) and ITS2 (5′-GCTGCGTTCTTCATCGATGC-3′). DNA libraries were constructed using the TruSeq Nano DNA LT Library Prep Kit (Illumina, Inc., San Diego, CA, USA). Qualified libraries were subjected to 250bp paired-end sequencing on an Illumina NovaSeq platform. Raw paired-end reads were processed using the DADA2 pipeline implemented in QIIME2 (v2022.11). Primer sequences were first removed using qiime cutadapt trim-paired, and reads without matched primers were discarded. Quality filtering, denoising, paired-end read merging, and chimera removal were subsequently performed using qiime dada2 denoise-paired. After denoising, amplicon sequence variants (ASVs) and the corresponding feature table were generated, and singleton ASVs occurring only once across all samples were removed. Taxonomic assignment was conducted in QIIME2 using the classify-sklearn method with a pre-trained Naive Bayes classifier. Bacterial 16S rRNA gene sequences were assigned using the SILVA database (release 138.1), whereas fungal ITS sequences were assigned using the UNITE database (release 9). Prior to α-diversity analysis, the ASV table was rarefied using qiime feature-table rarefy, with the rarefaction depth set to 95% of the sequence count of the sample with the lowest sequencing depth. α-Diversity was evaluated using the Chao1, Shannon, and Simpson indices.

### 4.5. Statistical Analyses

All figures were generated using Origin 2021 and R software (version 4.4.1), and the data were organized using Microsoft Excel 2011. SPSS 27.0.1 was used to perform one-way analysis of variance (ANOVA) on leaf functional traits and soil properties of *C*. *microphylla* across different restoration years, followed by Fisher’s least significant difference (LSD) post hoc test for multiple comparisons. Statistical significance was set at *p* < 0.05. Pearson correlation analysis was conducted to examine relationships among leaf functional traits, soil properties, and soil microbial diversity. To account for multiple comparisons, *p* values were adjusted using the false discovery rate (FDR) correction method. Redundancy analysis (RDA) was conducted to explain the overall variation in leaf functional traits in relation to soil properties and soil microbial communities. Monte Carlo permutation tests (999 permutations) were conducted to assess the significance of explanatory variables in the RDA model.

Partial least squares path modeling (PLS-PM) was used to examine the direct and indirect associations of restoration years, soil microbial communities, and soil properties with leaf functional trait using plspm R package. The analysis was based on 15 independent plot-level observations, comprising three replicate plots for each of the five restoration ages. Measurements from subsamples within each plot were aggregated to plot-level values before analysis. The model comprised five blocks: restoration age; bacterial community composition (*Sphingomonas*, *Nitrospira*, and *Bradyrhizobium*); fungal community composition (*Fusarium*, *Penicillium*, and *Mortierella*); soil properties (SOC, STN, STP, pH, and SWC); and leaf functional traits (SLA, LTD, LDMC, LNC, LCC, and LPC). The observed variables included in these blocks were selected according to their ecological relevance to plant–soil relationships and the results of the preceding correlation, redundancy, and collinearity analyses. Variables showing severe collinearity (VIF > 10) were excluded before model construction. All observed variables were standardized before model fitting. The statistical significance of the inner-model path coefficients was evaluated using two-sided *t*-tests based on the estimated standard errors from regressions fitted to the PLS-PM composite scores. Indirect associations were calculated as the products of the relevant path coefficients. Given the limited sample size, the PLS-PM analysis was considered exploratory, and all path coefficients were interpreted as measures of statistical associations.

## 5. Conclusions

This study investigated the variation in leaf functional traits and soil microbial communities, as well as their potential interactions, across different restoration years of *C*. *microphylla* plantations in an alpine sandy ecosystem. The main conclusions are as follows: (1) Leaf functional traits of *C. microphylla* exhibited distinct stage-dependent responses along the restoration chronosequence. Specifically, SLA, LA, and LT generally showed a decreasing–increasing pattern, whereas LDMC and LTD displayed an opposite trend. These coordinated trait changes indicate stage-dependent variation in leaf resource-use characteristics, with later restoration stages exhibiting trait combinations more consistent with relatively acquisitive characteristics. (2) Fungal α-diversity generally increased with restoration years, whereas bacterial Shannon and Simpson indices exhibited a unimodal pattern. These contrasting patterns suggest that bacteria and fungi may play differentiated ecological roles and possess distinct adaptive strategies during the restoration of alpine sandy ecosystems. (3) Soil properties were strongly associated with variation in plant leaf functional traits and soil microbial communities. Microbial communities were statistically associated with leaf functional traits both directly and indirectly through their relationships with soil conditions. These findings highlight the coordinated variation among plant, soil, and microbial components during alpine sandy ecosystem restoration and provide insights into the ecological processes associated with ecosystem functional recovery.

## Figures and Tables

**Table 1:** Soil properties of *C. microphylla* plantations along different restoration years.

Restoration Years	pH	EC (μS·cm^−1^)	SBD (g·cm^−3^)	SWC (%)	SOC (g·kg^−1^)	SIC (g·kg^−1^)	STN (g·kg^−1^)	STP (g·kg^−1^)
5 Y	7.74 ± 0.59 a	47.73 ± 8.41 b	1.42 ± 0.09 a	4.03 ± 0.21 a	0.99 ± 0.09 d	11.55 ± 0.95 a	0.10 ± 0.01 c	0.45 ± 0.03 ab
10 Y	7.72 ± 0.57 a	71.54 ± 1.58 a	1.36 ± 0.14 a	1.49 ± 0.30 c	2.12 ± 0.28 a	8.47 ± 1.17 b	0.21 ± 0.03 a	0.53 ± 0.07 a
15 Y	7.56 ± 0.10 a	39.75 ± 4.80 b	1.40 ± 0.12 a	1.23 ± 0.49 c	1.38 ± 0.04 c	8.59 ± 1.00 b	0.17 ± 0.01 b	0.37 ± 0.01 bc
23 Y	7.96 ± 0.48 a	41.92 ± 1.02 b	1.29 ± 0.15 a	2.59 ± 0.39 b	1.76 ± 0.28 b	9.72 ± 0.41 b	0.21 ± 0.02 a	0.36 ± 0.01 bc
36 Y	7.86 ± 0.04 a	61.92 ± 12.07 a	1.43 ± 0.05 a	2.15 ± 0.60 b	2.01 ± 0.13 ab	9.54 ± 0.47 b	0.23 ± 0.01 a	0.34 ± 0.10 c

Different lowercase letters in the same column indicate significant difference between different restoration years at 0.05 level. Data are presented as mean ± standard deviation (SD).

**Table 2:** Overview of sample plots along different restoration years.

Restoration Years	Elevation (m)	Total Community Cover (%)	Dominant Species Cover (%)	Mean Shrub Height (m)	Mean Basal Diameter (cm)	Crown Width (m)	
						North–South	East–West
5 Y	2876.30	37.67 ± 9.71 b	0.33 ± 0.10 b	1.10 ± 0.11 c	1.09 ± 0.11 b	1.20 ± 0.25 c	1.13 ± 0.29 c
10 Y	2913.07	53.00 ± 5.00 b	0.42 ± 0.03 b	1.08 ± 0.12 c	1.04 ± 0.03 b	1.16 ± 0.17 c	1.21 ± 0.22 bc
15 Y	2878.00	75.33 ± 7.51 a	0.68 ± 0.10 a	1.28 ± 0.27 c	0.91 ± 0.13 b	1.16 ± 0.25 c	1.35 ± 0.15 bc
23 Y	2870.87	90.00 ± 11.31 a	0.69 ± 0.01 a	1.62 ± 0.20 b	1.56 ± 0.28 a	2.44 ± 0.42 a	2.16 ± 0.42 a
36 Y	2834.77	78.33 ± 15.37 a	0.73 ± 0.08 a	2.02 ± 0.10 a	1.07 ± 0.14 b	1.73 ± 0.08 b	1.70 ± 0.17 ab

Different lowercase letters in the same column indicate significant difference among restoration years at 0.05 level. Data are presented as mean ± standard deviation (SD).

## Data Availability

The data set supporting the findings of this study are available from the corresponding author upon reasonable request.
